# Tetrahedral framework nucleic acids‐based delivery of MicroRNA‐22 inhibits pathological neovascularization and vaso‐obliteration by regulating the Wnt pathway

**DOI:** 10.1111/cpr.13623

**Published:** 2024-03-03

**Authors:** Xinyu Liu, Xiaoxiao Xu, Yanting Lai, Xiaodi Zhou, Limei Chen, Qiong Wang, Yili Jin, Delun Luo, Xiaoyan Ding

**Affiliations:** ^1^ State Key Laboratory of Ophthalmology, Zhongshan Ophthalmic Center Sun Yat‐sen University, Guangdong Provincial Key Laboratory of Ophthalmology and Visual Science Guangzhou China; ^2^ Innovative Institute of Chinese Medicine and Pharmacy Chengdu University of Traditional Chinese Medicine Chengdu China

## Abstract

The objective of this study was to investigate the effects and molecular mechanisms of tetrahedral framework nucleic acids‐microRNA22 (tFNAs‐miR22) on inhibiting pathological retinal neovascularization (RNV) and restoring physiological retinal vessels. A novel DNA nanocomplex (tFNAs‐miR22) was synthesised by modifying microRNA‐22 (miR22) through attachment onto tetrahedral frame nucleic acids (tFNAs), which possess diverse biological functions. Cell proliferation, wound healing, and tube formation were employed for in vitro assays to investigate the angiogenic function of cells. Oxygen‐induced retinopathy (OIR) model was utilised to examine the effects of reducing pathological neovascularization (RNV) and inhibiting vascular occlusion in vivo. In vitro, tFNAs‐miR22 demonstrated the ability to penetrate endothelial cells and effectively suppress cell proliferation, tube formation, and migration in a hypoxic environment. In vivo, tFNAs‐miR22 exhibited promising results in reducing RNV and promoting the restoration of normal retinal blood vessels in OIR model through modulation of the Wnt pathway. This study provided a theoretical basis for the further understanding of RNV, and highlighted the innovative and potential of tFNAs‐miR22 as a therapeutic option for ischemic retinal diseases.

## Graphical abstract

We selected tetrahedral framework nucleic acids (tFNAs) as a vector for microRNA22‐3p (miR22) to synthesise a tetrahedral framework nucleic acids‐microRNA22 (tFNAs‐miR22) complex. We investigated the regulation of tFNAs‐miR22 on blood vessels in vivo and in vitro via Wnt pathway, as a therapeutic option for ischemic retinal diseases.

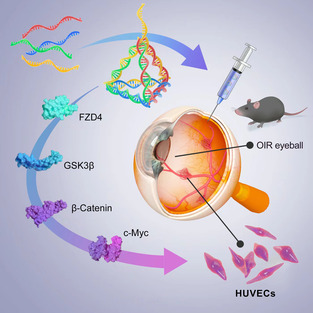

## 1 INTRODUCTION

Pathological retinal neovascularization is associated with prevalent blinding retinal diseases across all age groups, including retinopathy of prematurity (ROP), which currently constitutes the primary cause of acquired blindness in infants^1^, ^2^; proliferative diabetic retinopathy (PDR) as the leading cause of blinding eye diseases in middle‐aged adults^3^, ^4^; and retinal vein occlusion (RVO) as a significant contributor to blindness in the elderly.^5^ Hypoxia of retinal tissue in these conditions represents a critical pathological process, given that the retina, as one of the most metabolically active tissues in the human body, necessitates a continuous supply of oxygen. Consequently, retinal vascular disease impedes an adequate oxygen supply to the retinal tissue, leading to hypoxia and serving as a crucial link in the pathogenesis of pathological retinal neovascularization.^6^ In light of this, retinal laser photocoagulation has emerged as an essential treatment modality, based on the principle of reducing the oxygen demand of retinal tissue. However, this intervention alleviates retinal oxygen demand at the expense of local retinal tissue scarring, resulting in inevitable visual function impairment, such as visual field constriction.^7^, ^8^ Alternatively, intravitreal injection of anti‐VEGF drugs is currently the preferred regimen for the clinical management of retinal neovascular diseases.^3^, ^7^ Nonetheless, this approach also has limitations, such as inhibiting VEGF signal transduction, which may affect the growth of normal retinal vessels. Furthermore, after discontinuing regular injections, the retina remains in a state of ischemia and hypoxia, increasing the likelihood of recurrent pathological retinal neovascularization.^9^ Therefore, there is an urgent need to optimise treatment strategies for pathological retinal neovascularization.

The discovery of microRNAs (miRNAs) as key regulators of gene expression has revolutionised our understanding of cellular processes. These non‐coding RNAs have been postulated to play a direct or indirect role in the pathogenesis of retinal vascular disease.^10^ miRNA‐22‐3p (miR‐22), in particular, has been demonstrated to exhibit angiogenic effects in the central nervous system and other systemic systems, such as cerebral ischemia–reperfusion‐related diseases.^11^, ^12^, ^13^ Furthermore, miR22 has been shown to activate the Wnt/β‐catenin pathway or directly bind c‐Myc, thereby exerting biological effects and regulating the cell cycle.^14^, ^15^ Consequently, it is plausible to speculate that miR22 could promote retinal angiogenesis by selectively activating the Wnt pathway. However, due to the instability of miRNAs in vivo, the utilisation of miR22 as a therapeutic target for the treatment of retinal vascular diseases has not yet been investigated. Consequently, the development of efficient and safe delivery methods for miRNAs into the body has become a top priority for relevant scientific research.

Tetrahedral framework nucleic acids (tFNAs), as innovative three‐dimensional nucleic acid nanomaterials, exhibit biocompatibility, structural stability, and programmability, making them highly suitable for applications in drug delivery and biomedical therapy.^14^, ^16^, ^17^, ^18^, ^19^, ^20^ Our previous research demonstrated that tFNAs effectively inhibited pathological retinal neovascularization and abrogated physiological retinal vascular occlusion in an oxygen‐induced retinopathy (OIR) model through the PI3K/AKT/mTOR/S6K signalling pathway.^21^ Consequently, in this study, we selected tFNAs as a vector for miR‐22‐3p to synthesise a tFNAs‐miR22 complex. We anticipate that tFNAs will deliver miR‐22 stably to the retina and elicit their angiogenic and endothelial protective effects.

Thus, we endeavoured to implement tFNAs‐miR22 in human umbilical vein endothelial cells (HUVECs) to regulate the process of vascular formation and examined its therapeutic effects and potential mechanisms in a mouse model of OIR. Our findings indicate that tFNAs‐miR22 potently inhibits angiogenesis in HUVECs under hypoxic conditions. Intriguingly, in the OIR mouse model, tFNAs‐miR22 not only curtailed the development of pathological neovascularization but also diminished the size of the retinal avascular zone and fostered the complete maturation of physiological blood vessels, suggesting that it might serve as an efficacious therapeutic agent for retinal vascular diseases.

## 2 METHODS

All materials and methods are described in detail in Data S1: Supporting Information (Methods).

## 3 RESULTS

### 3.1 Synthesis and characterisation of tFNAs‐miR22

**FIGURE 1:**
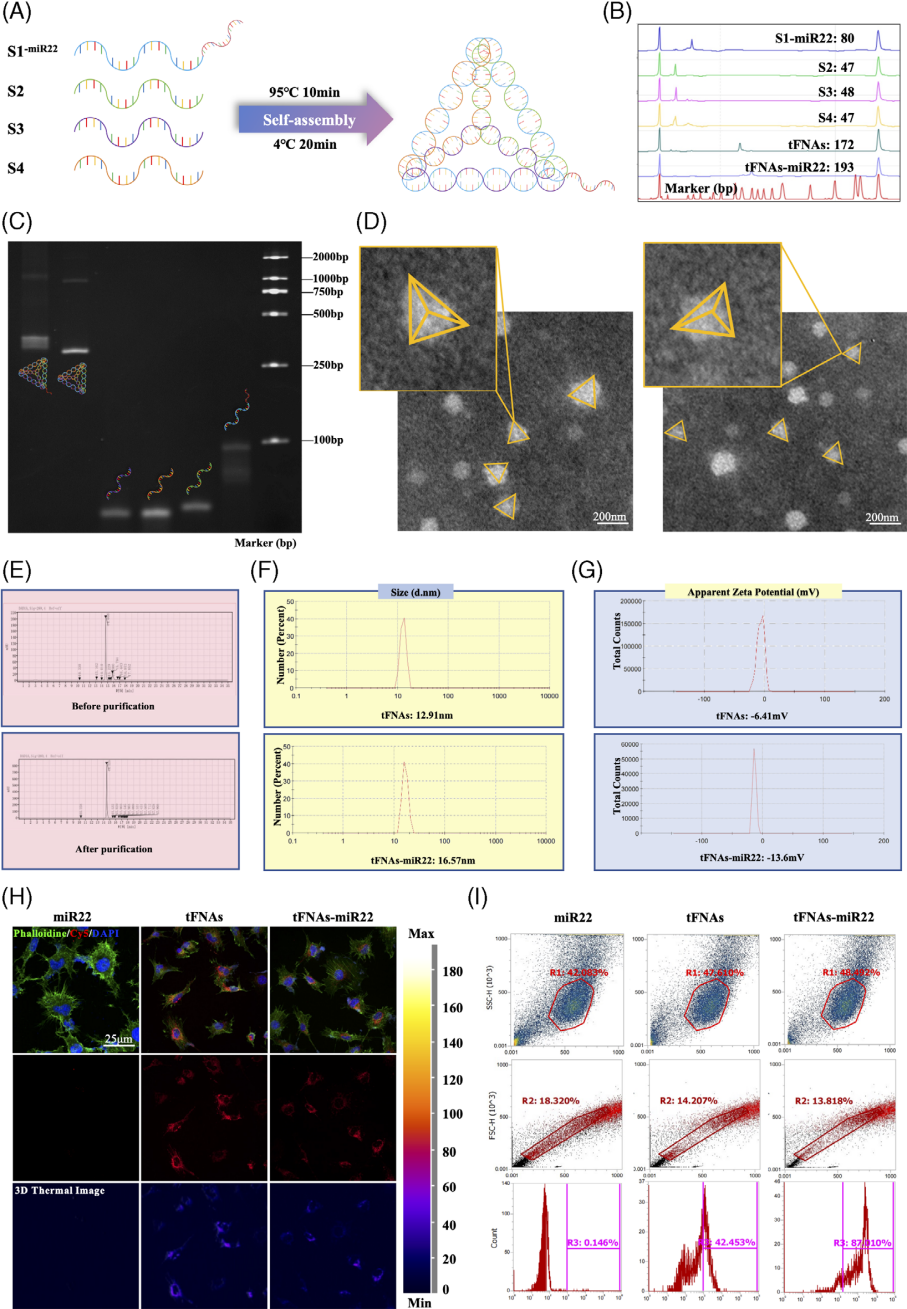
Synthesis and characterisation of tFNAs‐miR22. (A) The synthesis of tFNAs‐miR22 is illustrated in a schematic diagram. (B) Successful generation of tFNAs and tFNAs‐miR22 was confirmed using high‐performance capillary electrophoresis (HPCE). (C) Polyacrylamide gel electrophoresis (PAGE) was used to detect the molecular weights of the synthesised tFNAs and tFNAs‐miR22. (D) Transmission electron microscope (TEM) image shows the molecular structure of the synthesised tFNAs and tFNAs‐miR22, with scale bars measuring 200 nm. (E) High‐performance liquid chromatography (HPLC) was performed on purified tFNAs to remove mismatched bases or single strands, as shown in the results. (F) Dynamic light scattering (DLS) analysis characterises the properties of both tFNAs and tFNAs‐miR22. (G) Zeta potential analysis measures the stability of both tFNAs and tFNAs‐miR22. H. After 8 h, HUVECs show uptake of Cy5‐loaded‐tFNAS‐miR22, indicated by red fluorescence, while blue fluorescence represents nuclei staining and green fluorescence represents cytoskeleton staining. Scale bars measure 25 μm. (I) Flow cytometry is used to observe uptake of miR‐22 without transfection reagent as well as uptake of tFNAs‐miR22 by HUVECs.

The successful production of equimolar quantities of ssDNA, as indicated in Table S1, was accomplished under the name tFNAs‐miR22. Each single‐stranded DNA molecule was then divided into three smaller fragments that self‐assembled to form a triangular structure. These fragments were designed to hybridise with the other three strands through highly specific complementary base pairing. A visual representation of the synthesis process for tFNAs‐miR22 can be found in Figure 1A. In the single‐stranded DNA molecules, we engineered the S1 strand to contain the miR‐22 sequence, while incorporating Cy5, a fluorescent molecule, into the S2 strand for visualising the localization of tFNAs‐miR22 in subsequent experimental analyses. To confirm the formation of tFNAs and tFNAs‐miR22, HPCE analysis was conducted and confirmed that four ssDNA molecules were present, aligning with previous theoretical values (Figure 1B). The successful synthesis of tFNAs‐miR22 was further validated by performing 8% PAGE analysis, which yielded consistent results with those obtained from HPCE (Figure 1C). Additionally, we utilised transmission electron microscopy (TEM) to examine the structural characteristics of tFNAs‐miR22 (Figure 1D). The results revealed that the synthesised tFNAs‐miR22 exhibited a triangular shape, similar to previous findings. After synthesis, we isolated tFNAs‐miR22, and HPLC results before and after purification of tFNAs demonstrated the elimination of chromatographic heterogeneous peaks after the removal of mismatched bases or single strands (Figure 1E). The particle sizes of tFNAs and tFNAs‐miR22 were approximately 12.91 and 16.57 nm, respectively. As nucleic acids are negatively charged, their zeta potentials were −6.41 mV and −13.6 mV, respectively (Figure 1F, G), confirming the stability of the synthesised tFNAs and tFNAs‐miR22.

### 3.2 Cellular uptake of tFNAs‐miR22

Verification of tFNAs‐miR22 entry into HUVECs was conducted. HUVECs were incubated with Cy5‐loaded tFNAs‐miR22 for a period of 24 h. The intracellular localization of tFNAs‐miR22 was determined by immunofluorescence assay, revealing its presence in the cytoplasm (Figure 1H). Cells were subsequently collected for flow cytometry analysis following 24 hours of treatment with Cy5‐loaded tFNAs‐miR22. This analysis demonstrated that tFNAs‐miR22 had entered 87.010% of the cells, whereas the entry rate for the control group was only 0.146% (*p* < 0.001, Figure 1I).

### 3.3 tFNAs‐miR22 inhibit HUVECs cell proliferation, tube formation, and migration in vitro

**FIGURE 2:**
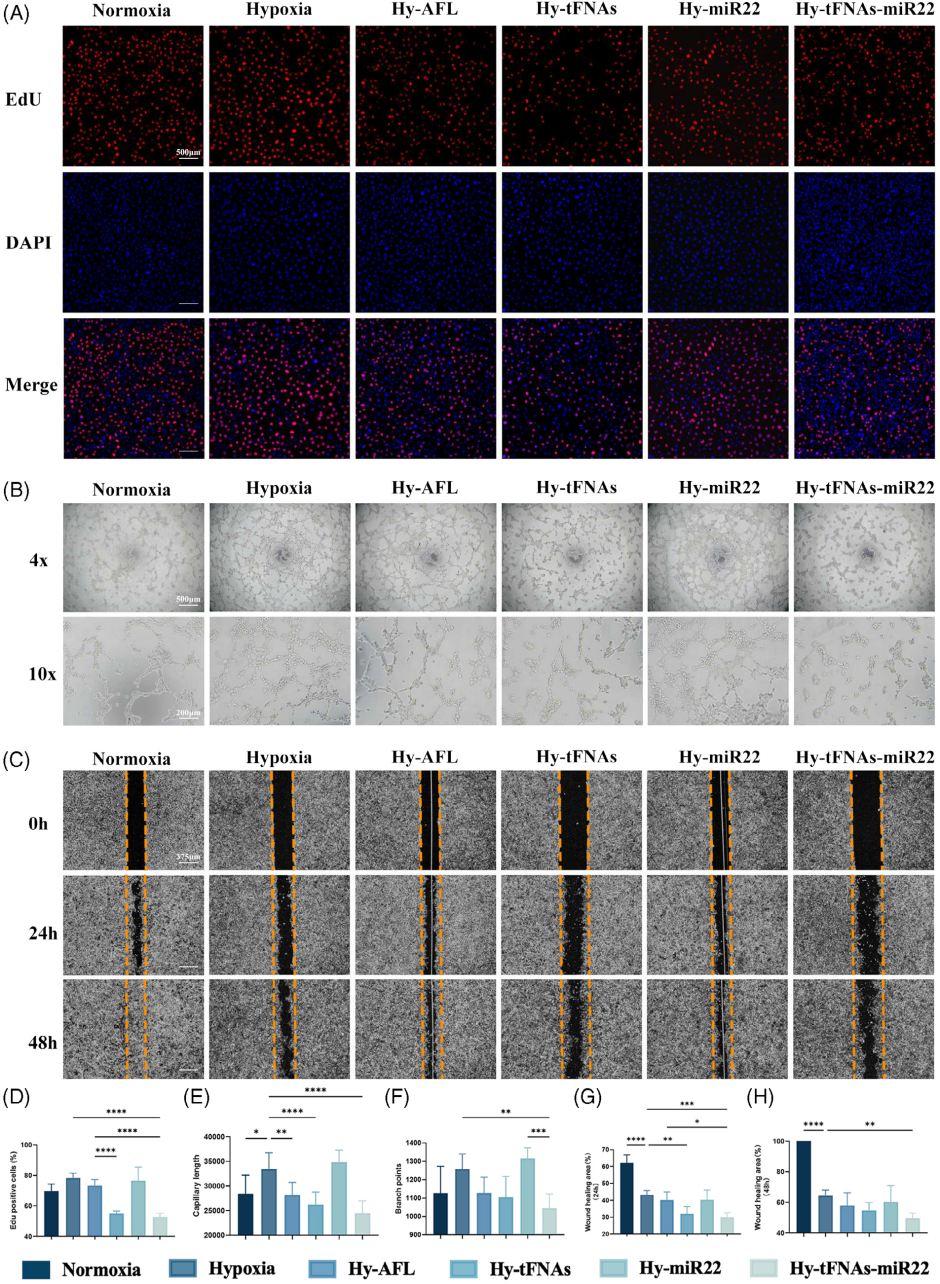
tFNAs‐miR22 inhibit angiogenesis in vitro. (A) The cell proliferation assay results of HUVECs after various treatments were evaluated. The scale bars used in the images are 500 μm. (B) Tube formations of HUVECs were examined at 12 h following different treatments. The scale bars used in the images are 500 and 200 μm. (C) Binary Image analysis was performed on scratch‐wound assays using HUVECs at 0, 24, and 48 h post‐treatment to assess wound healing progress. The scale bars used in the images are 375 μm. (D) The percentage of nuclei that were positive for Hoechst (blue) and also colocalized with EdU (red) was determined through quantification analysis. The data are presented as the mean ± standard deviation (*n* = 6). (E) Statistical analysis was performed on the measurements of capillary lengths, and the data are presented as the mean ± SD (*n* = 6). (F) Branch points were quantified and analysed statistically to evaluate angiogenesis potential. Data are presented as mean ± SD (*n* = 6). (G) and (H) Wound healing area rates at both 24 and 48 h post‐treatment were calculated. Data are presented as mean ± SD (*n* = 6). Statistical analysis: *p < 0.05, **p < 0.01, ***p < 0.001, and ****p < 0.0001 result from ANOVA test.

First, HUVECs were incubated with a vehicle or 1 μg/μL aflibercept (AFL) or 100 nmol/L tFNAs, miR‐22, or tFNAs‐miR22 for 24 h under normoxic or hypoxic conditions (37°C, 1% O_2_, 5% CO_2_). Cell proliferation was assessed using the EdU assay (Figure 2A). The findings revealed that the proliferation activity of HUVECs was significantly reduced in tFNAs (55.08% ± 1.53%) and tFNAs‐miR22 (52.99% ± 2.24%) compared to the control group (78.42% ± 3.13%) in a hypoxic culture environment, with tFNAs‐miR22 demonstrating the most prominent effect (Figure 2D). These results suggest that treatment with tFNAs‐miR22 significantly mitigates hypoxia‐induced cell proliferation.

We next cultured HUVECs for 24 h and examined tube formation under normoxic and hypoxic conditions (Figure 2B). The findings revealed that the capillary length and branch points of HUVECs under hypoxic conditions (33,473 ± 3280 and 1257 ± 82.67) exhibited a microscopic increase compared to the normoxic group (28,386 ± 3834 and 1126 ± 146.2) (Figure 2E, F), indicating that hypoxic conditions promote enhanced cell tube formation. Notably, AFL treatment (28,206 ± 2515 and 1127 ± 86.18), tFNAs treatment (26,220 ± 2516 and 1105 ± 113.2), or tFNAs‐miR22 treatment (24,495 ± 2492 and 1045 ± 77.18) significantly diminished their counts (Figure 2G, H), with tFNAs‐miR22 treatment exhibiting the most significant reduction. These results suggest that tFNAs‐miR22 exhibits potent inhibitory effects on HUVECs tube formation functionality.

The migration capacity of HUVECs at 24 and 48 h was further assessed using a scratch assay (Figure 2C). The findings revealed that the wound healing area ratio of HUVECs cultured under hypoxic conditions at 24 and 48 h (43.31% ± 2.52% and 64.45% ± 3.61%) was significantly reduced compared to the normoxic group (62.11% ± 4.75% and 100.00% ± 0.00%) under the microscope (Figure 2G, H). However, a quantitative analysis of the wound healing area ratio under hypoxic conditions demonstrated that cell migration was significantly impaired in cells treated with tFNAs‐miR22 (29.97% ± 2.63% and 49.63% ± 3.37%) compared to AFL‐treated (40.18% ± 4.66% and 58.01% ± 8.23%), tFNAs‐treated (31.88% ± 4.35% and 54.68% ± 5.25%), or miR22‐treated (40.23% ± 5.87% and 60.32% ± 10.64%) cells (Figure 2G, H), suggesting that tFNAs‐miR22 exhibits a more prominent ability to inhibit HUVECs migration compared to other treatment groups.

### 3.4 tFNAs‐miR22 reduces angiogenesis and inhibits the VO in OIR model

**FIGURE 3:**
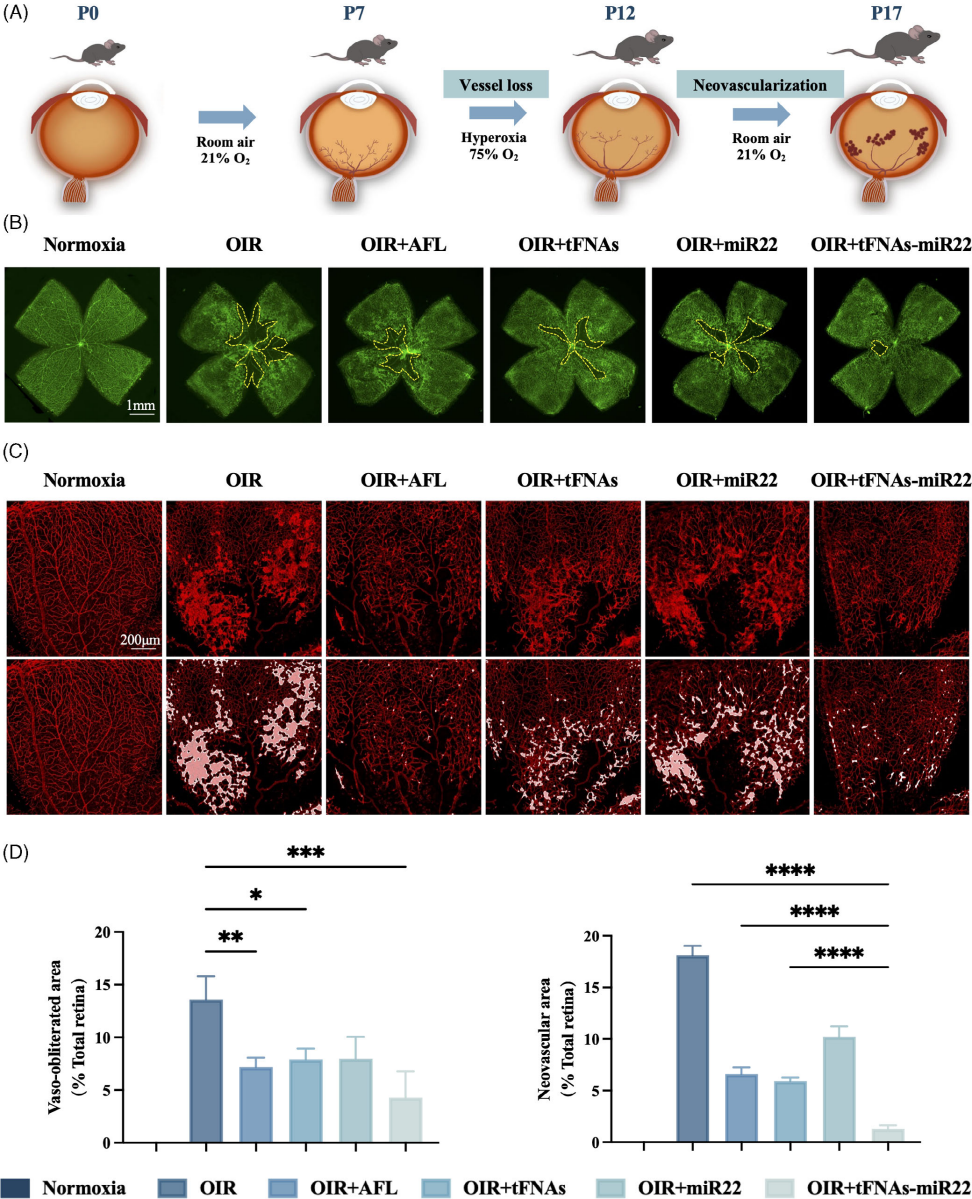
tFNAs‐miR22 reduce avascular area and inhibit pathological angiogenesis in OIR model on retinal flat‐mount. (A) Explanation of the in vivo experiment using the OIR model to study retinal anatomy. OIR refers to oxygen‐induced retinopathy. (B) Illustrative images of the entire retinal tissue stained with isolectin B4 (IB4). The vaso‐obliterated area (VO) is indicated by a yellow dotted line. The scale bars represent 1 mm. (C) Representative visuals displaying the neovascular area. Scale bars: 1 mm. The second row highlights the neovascular region, depicted by a white line. (D) Assessment of both vaso‐obliterated area (VO) and neovascular area. The data are presented as mean ± SD (*n* = 3). Statistical analysis: the ANOVA test was applied, ***p ≤0.001, **p ≤0.01, and *p <0.05.

The OIR model was successfully replicated in C57BL/6J mice. Subsequently, intravitreal administration of vehicle (1 μL), AFL (10 μg/μL), tFNAs (1 μmol/L), miR22 (1 μmol/L), and tFNAs‐miR22 (1 μmol/L) was performed at postnatal day 12 (P12) to investigate the potential therapeutic efficacy of tFNAs‐miR22 treatment (Figure 3A). We assessed the inhibitory effects of these drugs on avascular area formation and retinal neovascularization (Figure 3B, C). Mice maintained under normoxic conditions (0.00% ± 0.00% and 0.00% ± 0.00%) were used as controls, while OIR mice in the vehicle‐treated groups (13.59% ± 2.197% and 18.52% ± 0.94%) exhibited significantly enlarged avascular areas and increased retinal neovascularization, confirming successful modelling of OIR mice (*p* < 0.0001).

**FIGURE 4:**
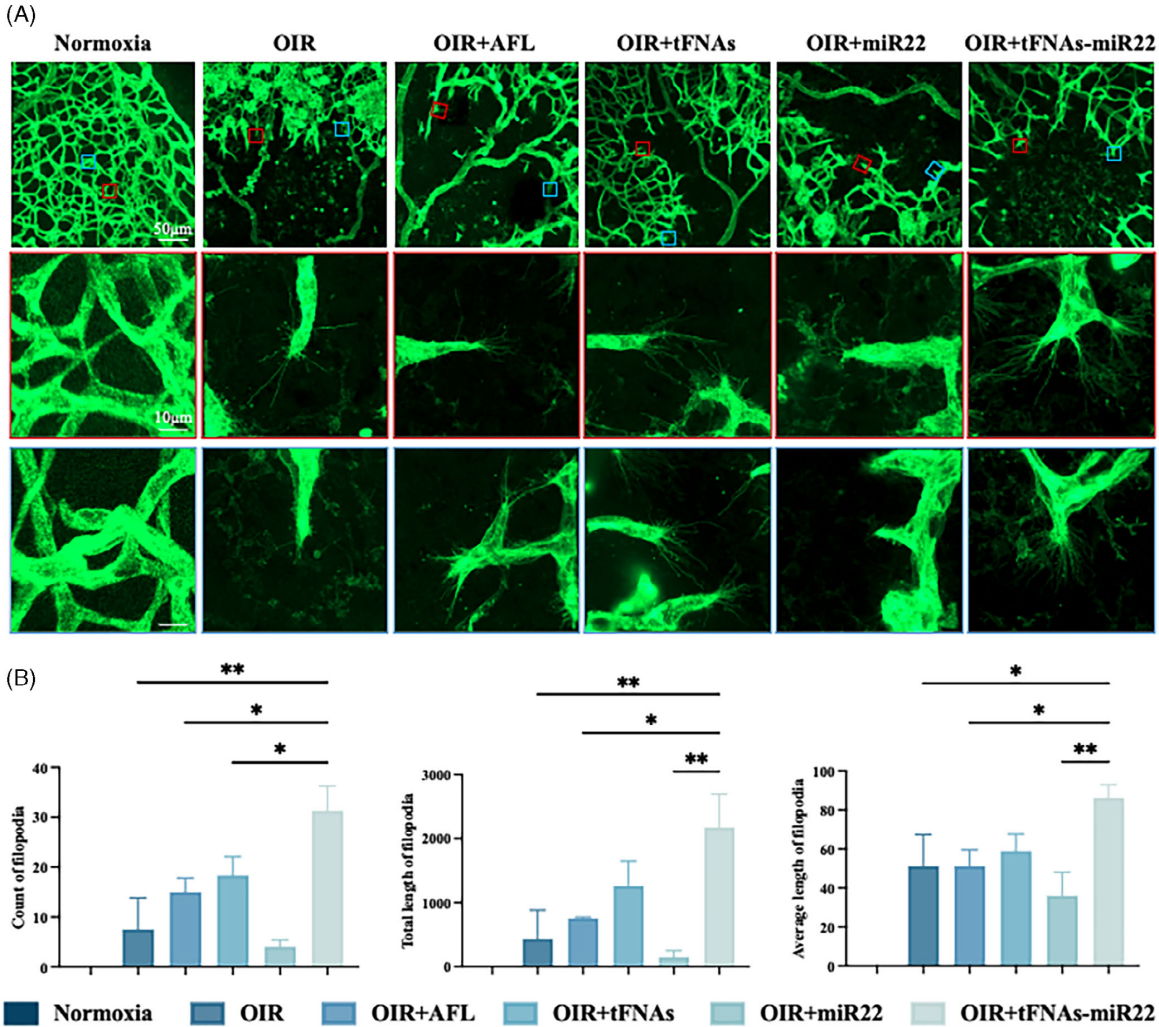
tFNAs‐miR22 reduce the number and mean length of filopodia in OIR model on retinal flat‐mount. (A) Representative images of filopodia (dashed boxes in the second row) from Normoxia, OIR, OIR + AFL, OIR + tFNAs, OIR + miR22, and OIR + tFNAs‐miR22 retinas. The magnified images of filopodia in the second and bottom row. (Red line and blue line). Scale bars are: 50 μm (Top row); 10 μm (second row); 10 μm (Bottom row). (B) Analysis of count, total lengths and average length of filopodia. Data are presented as mean ± SD (*n* = 6). All error bars represent SEM. Statistical analysis: *p < 0.05, **p < 0.01, ***p < 0.001, and ****p < 0.0001 result from ANOVA test.

The results demonstrated that AFL treatment (7.18% ± 0.91% and 6.74% ± 0.66%), tFNAs treatment (7.89% ± 1.05% and 6.05% ± 0.36%), miR22 treatment (7.97% ± 2.09% and 10.46% ± 1.02%), and tFNAs‐miR22 treatment (4.29% ± 2.50% and 1.33% ± 0.34%) exhibited efficacy in reducing the area of avascular zone and retinal neovascularization in the OIR mouse model compared to the vehicle group (13.59% ± 2.197% and 18.52% ± 0.94%) (Figure 3D). Notably, tFNAs‐miR22 treatment showed the most significant therapeutic effect among all groups, surpassing other treatments. In addition, we focused on the extension characteristics of filopodia at the leading edge of growing vessels located at the avascular zone of the retina by employing confocal microscopy at high magnifications (Figure 4A). Compared to the vehicle group in the OIR model (7.50 ± 6.36 and 51.19 ± 16.21), we observed a significant increase only in the number (31.33 ± 4.93), total length (2174.00 ± 519.70) and mean length (86.27 ± 6.64) of filopodia in the tFNAs‐miR22‐treated group, while no significant changes were observed in other treatment groups including AFL‐treated group (Figure 4B).

### 3.5 tFNAs‐miR22 ameliorate cell proliferation, and migration and reduce RNV via the Wnt pathway

**FIGURE 5:**
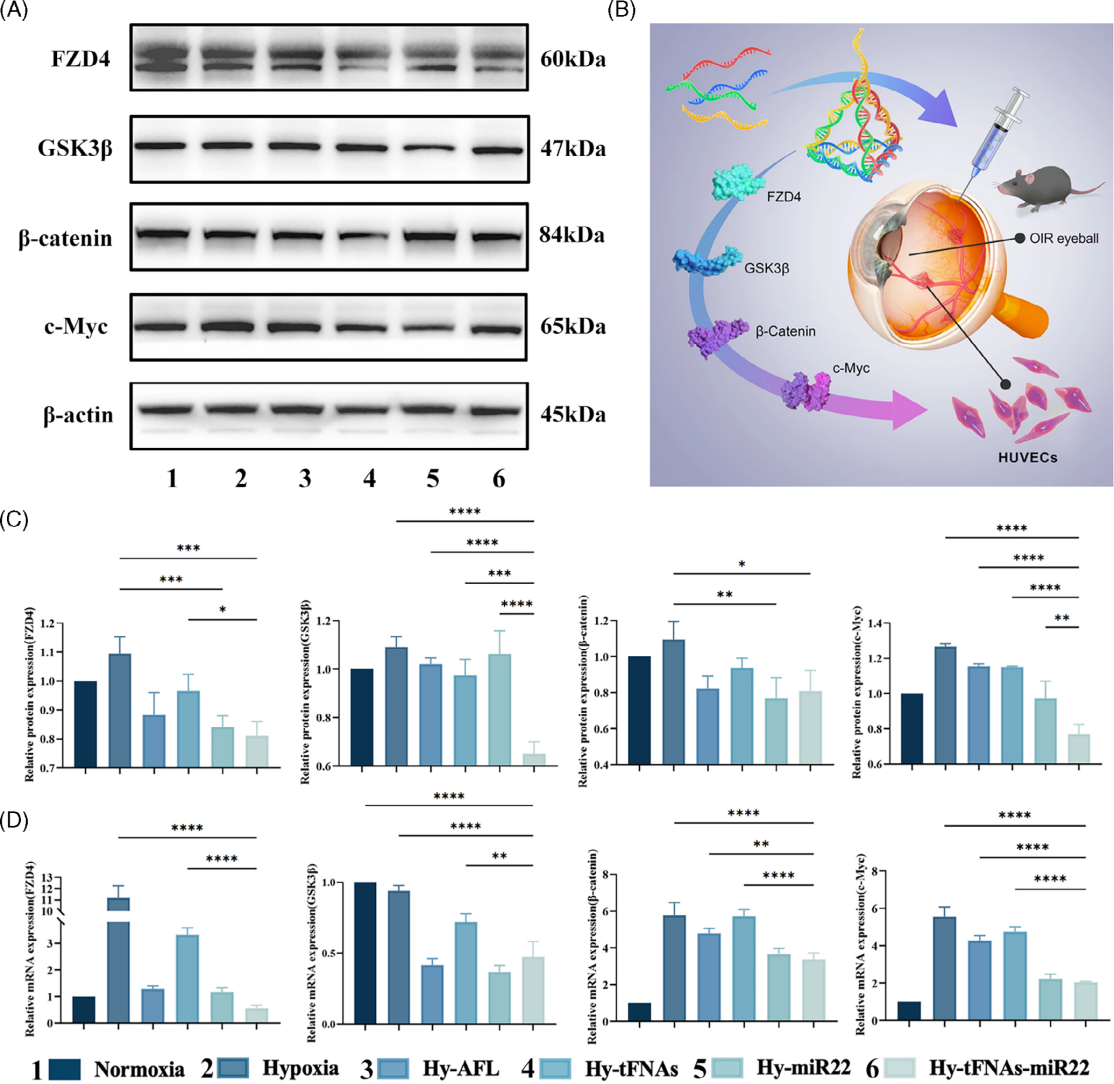
tFNAs‐miR22 prevent HUVECs proliferation and reduce avascular area via the Wnt pathway. (A) The expression levels of FZD4, GSK3β, β‐catenin, and c‐Myc were analysed using Western blotting (with β‐Actin as the internal control). (B) A summary diagram illustrating the synthesis process of tFNAs‐miR22 and its role in reducing retinal vascular abnormalities through the Wnt pathway. (C) The protein expression intensity of FZD4, GSK3β, β‐catenin, c‐Myc, and β‐actin was quantified relative to their respective controls. Mean ± SD values are presented (*n* = 3). (D) The mRNA expression intensity of FZD4, GSK3β, β‐catenin, c‐Myc, and β‐actin was measured relative to their respective controls. Mean ± SD values are presented (*n* = 3).Statistical analysis: *p < 0.05, **p < 0.01, ***p < 0.001, and ****p < 0.0001 result from ANOVA test.

To confirm the impact of tFNAs‐miR22 on cell proliferation and migration through the Wnt pathway in this study, we assessed the relative expression levels of FZD4, GSK3β, β‐catenin, c‐Myc protein, and mRNA in HUVECs under normoxic or hypoxic conditions using WB and qPCR techniques (Figure 5). Our WB results revealed that FZD4, GSK3β, β‐catenin, and c‐Myc protein expression levels were significantly upregulated under hypoxia (*p* < 0.05), but markedly decreased after treatment with tFNAs‐miR22 compared to control (*p* < 0.005). Consistently, our qPCR results demonstrated a similar trend where tFNAs‐miR22 effectively suppressed mRNA expression of FZD4, GSK3β, β‐catenin, and c‐Myc. Interestingly, transfection with miR22 resulted in inhibition of HUVECs function following tFNAs‐miR22 treatment; highlighting the crucial role played by miR22 in modulating the Wnt pathway.

## 4 DISCUSSION

RNV is a prominent aetiology of irreversible visual impairment and blindness in millions of patients worldwide, encompassing conditions such as ROP, PDR, and RVO.^22^, ^23^, ^24^, ^25^, ^26^, ^27^ However, the use of VEGF inhibitors and photocoagulation therapy presents potential drawbacks due to complications arising from VEGF signalling blockage, persistent ischemic conditions, and loss of retinal tissue.^28^, ^29^, ^30^, ^31^, ^32^, ^33^ This highlights an urgent need for safer and more durable and effective treatment options that minimise the need for frequent interventions for RNV‐related diseases.

In our study, we have successfully synthesised tFNAs‐miR22, as evidenced by comprehensive analyses including TEM, HPCE, DLS, and zeta potential analysis. The exceptional cellular uptake of tFNAs‐miR22 was confirmed by immunofluorescence and flow cytometry assays. Aligning with previous studies, DNA nanomaterials exhibited remarkable stability and various biological applications.^20^, ^34^, ^35^ The most notable characteristic of tFNAs, in comparison to ssDNA and other spatial nanostructures, is its significantly enhanced endocytosis capability. Importantly, tFNAs have been observed to penetrate the cell membrane without the assistance of transfection agents. Furthermore, Fan et al. were the first to report that Cy3‐labelled tFNAs adjust their orientation by attaching their corners to the cell membrane, thereby minimising charge repulsion and facilitating charge redistribution. This attachment is subsequently followed by caveolin‐mediated pathways for endocytosis of tFNAs and their entry into lysosomes in a microtubule‐dependent manner.^36^, ^37^, ^38^ Diverging from previous investigations on miRNA‐loaded tFNAs,^39^, ^40^ our approach included a purification process for tFNAs‐miR22 to eliminate excessive and mismatched bases, as well as single strands, followed by verification using HPLC. This purification step significantly bolsters the reliability of tFNAs‐miR22 for future pharmacodynamic and mechanistic investigations.

HUVECs, as a well‐established cellular model for investigating angiogenesis, demonstrate proliferation, tube formation, and migration under hypoxic conditions, mimicking the pathological process of retinal neovascularization in vitro.^41^, ^42^ In our study, we demonstrated that tFNAs, AFL, and tFNAs‐miR22 effectively suppressed these activated functions. The lack of efficacy observed in the miR22‐treated group can be attributed to the inherent limitations of miRNA transfection, including its susceptibility to degradation and variable transfection efficiency.^43^ Among these, tFNAs‐miR22 demonstrated the most substantial inhibitory effect, suggesting stable miR22 transfer into cells by tFNAs,^20^, ^34^, ^35^ and indicating a potential synergistic effect between miR22 and tFNAs in combating RNV. In parallel, the OIR mouse model, a cornerstone in anti‐angiogenic research, highlights vascular abnormalities such as RNV and vaso‐obliteration (VO).^44^ while AFL reduced retinal NV, it was less effective against VO.^45^ Contrastingly, in our study, tFNAs‐miR22 showed a significant advantage over AFL in inhibiting both RNV and VO. This finding underscores the potential of tFNAs‐miR22 as a more effective and comprehensive treatment approach for RNV‐related conditions.

The Wnt signalling pathway plays a pivotal role in governing both physiological and pathological intraocular vasculature.^46^ Impairment of Wnt signalling leads to delayed vascular pruning and secondary pathological vascular proliferation.^47^, ^48^, ^49^, ^50^, ^51^ Prior research has highlighted miR22's role in modulating the Wnt pathway, specifically by inhibiting c‐Myc, a key downstream effector.^14^, ^52^, ^53^ Therefore, we further investigated the mechanism underlying tFNAs‐miR22, focusing on its interaction with the Wnt pathway. Our study observed significant inhibition of mRNA and protein expression related to the Wnt pathway by tFNAs‐miR22 through qPCR and Western blotting experiments, confirming its inhibitory effect on retinal neovascularization via modulation of the Wnt pathway.

Our study demonstrated that tFNAs‐miR22 markedly outperformed in reducing the area of retinal VO in OIR models if compared with AFL. Notably, tFNAs‐miR22 significantly increased both the number and length of filopodia at the VO's periphery, a capability not observed with AFL. Given the pivotal role of filopodia extension in regulating angiogenic sprouting and guiding functional retinal vessel formation,^54^ our findings substantiated that tFNAs‐miR22 could facilitate physiological blood vessel development in VO areas. Our previous investigations demonstrated that tFNAs could facilitating physiological angiogenesis by regulating the Notch signalling pathway and PI3K/AKT/mTOR signalling pathway.^21^, ^55^, ^56^ Moreover, various studies have demonstrated miR22's protective effects on angiogenesis and vascular endothelial injury in cerebral ischemia–reperfusion disease models^13^, ^57^ and its role in maintaining physiological vascular development by influencing vascular smooth muscle cells' proliferation and migration.^58^ Considering these findings, we hypothesise a synergistic effect between miR‐22 and tFNAs in fostering physiological angiogenesis, particularly highlighting the potential of tFNAs‐miR22 in reducing retinal VO in the OIR model. However, further research is needed to fully elucidate the specific molecular mechanisms through which tFNAs‐miR22 promotes physiological vessel development in VO.

## 5 CONCLUSION

In summary, our research demonstrates the efficient and safe entry of tFNAs‐miR22 into vascular endothelial cells. It effectively inhibits the proliferation, migration, and tube formation of HUVECs in vitro, thereby suppressing pathological retinal neovascularization. In OIR mice, tFNAs‐miR22 not only mitigates abnormal blood vessel growth but also aids in restoring normal vasculature in ischemic retinas by reducing vaso‐obliteration. Our findings indicate that the primary mechanism of action for tFNAs‐miR22 is through modulation of the Wnt signalling pathway. This study highlights the innovative potential of tFNAs‐miR22 as a therapeutic option for ischemic retinal diseases, which are a leading cause of blindness worldwide in both developed and developing countries.

## AUTHOR CONTRIBUTIONS

X. Liu, Y. Lai, and X. Zhou contributed to this work in original draft and review & editing writing, formal analysis. X. Liu, Y. Lai, and Y. Jin contributed to data curation and formal analysis. X. Xu, L. Chen, Q. Wang, and Y. Jin contributed to methodology and software. D. Luo and X. Ding provided resources. X. Ding and Y. Lai provided validation for this article. X. Ding contributed to this work in funding acquisitions, writing and supervision.

## CONFLICT OF INTEREST STATEMENT

The authors declare no conflict of interest.

## Supporting information


**Data S1.** Supporting Information

## Data Availability

Research data are not shared.
