# Supplementary material for: Tetrahedral framework nucleic acids‐based delivery of MicroRNA‐22 inhibits pathological neovascularization and vaso‐obliteration by regulating the Wnt pathway
Source: Cell Prolif. 2024 Mar 3;57(7):e13623. doi: 10.1111/cpr.13623 (PMC11216936; doi:10.1111/cpr.13623)
Supplement: Supplementary file 1 — Data S1. Supporting Information [file CPR-57-e13623-s001.docx]

**METHODS**

1. **Synthesis of tFNAs-miR22**

Genescript (Nanjing, China) synthesized and identified four single-stranded DNAs (ssDNAs) listed in **Table 1**. A TM buffer at pH 8.0 was prepared with Tris-HCl and MgCl2, followed by combining equal concentrations of the four ssDNAs in the buffer with one end carrying microRNA22 (miR22). The resulting mixture was thoroughly mixed, centrifuged, heated to 95℃ for 10 minutes, and then cooled to 4℃ for 20 minutes to obtain tFNAs-miR22. The sequences of these ssDNA species are provided in Supplementary **Table 1**. After synthesis, a pre-purification sample of 100μL tFNAs-miR22 was introduced into an HPLC system for purification using different mobile phases. The tFNAs-miR22 samples were subjected to purification using a DNA PacTMPA100 chromatographic column (ThermoFisher Scientific, USA) at a flow rate of 10 mL·min-1. Purification was carried out using different mobile phases: mobile phase A consisted of 25mM Tris-HCL, while mobile phase B contained 25mM Tris-HCL and 375mM NaClO4 C. Finally, the collected main peak of the sample underwent HPLC analysis to assess its purity.

Supplementary **Table 1 The sequences of the four ssDNAs and microRNA22**

| ssDNA | Base sequence (5’-3’) |
| --- | --- |
| S1 | ATTTATCACCCGCCATAGTAGACGTATCACCAGGCAGTTGAGACGAACATTCCTAAGTCTGAA |
| S2-Cy5 | ACATGCGAGGGTCCAATACCGACGATTACAGCTTGCTACACGATTCAGACTTAGGAATGTTCG |
| S3 | ACTACTATGGCGGGTGATAAAACGTGTAGCAAGCTGTAATCGACGGGAAGAGCATGCCCATCC |
| S4 | ACGGTATTGGACCCTCGCATGACTCAACTGCCTGGTGATACGAGGATGGGCATGCTCTTCCCG |
| microRNA22 | AAGCUGCCAGUUGAAGAACUGU |

1. **Characterization of tFNAs-miR22**

We conducted experiments using the methodologies outlined in previous studies to validate the successful production of tFNAs-miR22. In summary, we utilized polyacrylamide gel electrophoresis (PAGE) and high-performance capillary electrophoresis (HPCE) techniques to identify differences in molecular weights between tFNAs-miR22 and its counterparts. Furthermore, a nanoparticle size analyzer was employed to confirm variations in zeta potential and particle sizes of the two reagents, thereby verifying the synthesis's success.

1. **Cell culture and treatment**

HUVECs were acquired from ATCC and cultured in 1 x DMEM/F-12 basic medium (Gibco, USA) supplemented with a complete antibiotic solution ( 10,000 μg·mL-1 streptomycin and 10,000 U mL-1 penicillin) (Gibco, USA) and 10% FBS (Gibco, USA). The cells were maintained under normoxia conditions at 37℃ for 24 hours. Following this, HUVECs were exposed to either normoxia (37℃, 5%CO_2_) or hypoxia (37℃, 1% O_2_, 5%CO_2_) along with different concentrations of treatment agents (vehicle control, tFNAS at a concentration of 100 nmol·L-1, or AFL at a concentration of 1μg·μL-1) in the original DMEM/F-12 medium containing appropriate levels of FBS for an additional day.

1. **Uptake of Cy5-loaded-tFNAs-miR22**

In this study, we examined the uptake of tFNAs-miR22 in HUVECs by incorporating Cy5 fluorescence into one of the ssDNAs (S2). HUVECs were cultured at a density of 4×10^5^ cells per well on a 6-well plate and allowed to incubate for 24 hours before being treated with Cy5-tFNAs-miR22 for another 24 hours. The flow cytometry analysis was performed using an Attune NxT instrument from ThermoF Scientific, USA, measuring the intracellular fluorescence intensity at 24 hours post-treatment after selecting suitable samples for analysis.

1. **Cell immunofluorescence assay**

The expression of proteins associated with the formation of new blood vessels and programmed cell death was assessed using immunofluorescence staining. Human umbilical vein endothelial cells (HUVECs) were cultured in 6-well plates (Corning, USA) at a density of 9.6×105 cells and subjected to the same treatment as described previously. Subsequently, the cells were fixed in PBS containing 4% paraformaldehyde (PFA) for 15 minutes, followed by permeabilization and blocking with a solution containing 0.5% Triton X-100 (Biofroxx, Germany) and 1% BSA (Biosharp, Anhui, China) for 20 minutes. After that, the samples were incubated overnight at a temperature of 4℃ with the primary antibody. Following this step, they were exposed to a secondary antibody against rabbit IgG [diluted at a ratio of 1:1000; catalog number:4413; Cell Signaling TECHNOLOGY(CST)] under room temperature conditions for a duration of two hours. Then, Hoechst 33342 dye (Solarbio, Beijing, China) was used to stain the cell nucleus while phalloidin dye (Solarbio, Beijing, China) was employed to label the cytoskeleton. Finally, anti-fluorescence quenching sealed tablets were utilized to fixate the samples.

1. **Real-time fluorescence quantitative PCR**

Real-time fluorescence quantitative PCR (RT-PCR) technology was utilized to assess the expression of relevant genes. Total RNA was extracted using TRNzol Universal (TIANGEN, Beijing, China), followed by purification and reverse transcription with the FastKing RT Kit With gDNase (TIANGEN, Beijing, China). The target mRNAs were amplified through quantitative RT-PCR using SYBR Green Realtime PCR Master Mix (TOYOBO, Shanghai, China). The primers for the genes (FZD4, GSK3β, β-catenin, c-Myc) were designed using a BLAST search and are listed in Table 2. The amplification of β-actin was used as a control during primer design.

**Table 2 The sequences of PCR primers**

| Gene | Primer sequence (5’-3’) |
| --- | --- |
| FZD4 | Forward：GTCTTTCAGTCAAGAGACGCTG  Reverse：GTTGTGGTCGTTCTGTGGTG |
| GSK3β | Forward：GGCAGCATGAAAGTTAGCAGA  Reverse：GGCGACCAGTTCTCCTGAATC |
| β-catenin | Forward：CTTACACCCACCATCCCACT  Reverse：CCTCCACAAATTGCTGCTGT |
| c-Myc | Forward：GGCTCCTGGCAAAAGGTCA  Reverse：CTGCGTAGTTGTGCTGATGT |

1. **Protein extraction and western blot analysis**

HUVECs were cultured and divided into various experimental groups. Following a 24-hour incubation period, protein was extracted from the HUVECs using RIPA buffer (Solarbio, Beijing, China) supplemented with protease inhibitors. The concentration of protein was determined using the BCA Protein Quantitation Assay Kit (KeyGEN, Nanjing, China) with a protein standard solution as a reference. Supernatant samples containing 20 μg of protein were subjected to heat treatment at 95℃ for 5 minutes and subsequently separated on SurePAGE™ Bis-Tris gel (GenScript, USA). The separated proteins were then transferred onto a polyvinylidene difluoride filter (PVDF) membrane (Millipore, Merck, Germany). After blocking with 5% defatted milk in TBS-Tween-20 (TBST) at room temperature for an hour, the PVDF membranes were incubated overnight at 4℃ with diluted primary antibodies: FZD4 (1:1000; PA5-142461; Invitrogen), GSK3β (1:1000; ET1607-71; Huaan Biotechnology), β-catenin (1:1000;14-2567-82; Invitrogen), c-Myc(1:1000; MA1-980; Invitrogen). Subsequently,the PVDF membranes underwent five washes with TBST before being further incubated with anti-rabbit IgG Horseradish peroxidase(HRP)-linked antibody(1:3000 ;7074,CST) at room temperature for two hours. Finally,the visualization of proteins was achieved using an enhanced chemiluminescence system(ProteinSimple ,USA).

1. **Angiogenesis experiments**
   1. **EdU cell proliferation assay**

To validate the inhibitory impact of tFNAs-miR22 on cellular proliferation, we employed the Alexa Fluor 488 Click-iT EdU Imaging Kits (ThermoFisher Scientific, USA) for the detection of 5-Ethynyl-2'-deoxyuridine (EdU) incorporation. HUVECs were seeded in 6-well plates and incubated for 24 hours prior to subjecting them to the treatments described in the 'Cell culture and treatment' section. The EdU cell proliferation assay was conducted according to the manufacturer's guidelines. Confocal laser microscopy (Carl Zeiss, Oberkochen, Germany) was utilized for visualizing fluorescence staining images of HUVECs within the 6-well plates. Statistical analysis was performed using ImageJ software. Each experiment was replicated a minimum of three times.

- 1. **Tube formation assay**

Tube formation experiments were performed to investigate the effect of tFNAs-miR22 on angiogenesis in HUVECs. HUVECs were cultured in 6-well plates for 24 hours. A pre-chilled 96-well plate was coated with Matrigel solution (50μL per well) and incubated at 37℃ for 30 minutes to allow gel formation. Then, HUVECs (approximately 1.2×10^5^ cells mL-1) with vehicle, HUVECs (approximately 1.2×10^5^ cells mL-1) mixed with tFNAs/miR22/tFNAs-miR22 (100 nmol L-1), and HUVECs (approximately 1.2×10^5^ cells mL-1) mixed with AFL (40 mg·mL-1, equivalent to a concentration of 1 μg·μL-1), were seeded onto the prepared gel-containing wells in the 96-well plate. The plate was then incubated under either normoxia conditions (37℃, 5%CO2) or hypoxia conditions (37℃, l% O2, 5% CO2) using the original DMEM/F-12 medium supplemented with FBS accordingly. Images of each group's wells were captured using an Inverted Biologic Microscope (IX2-SL, Olympus Corporation, Japan). Subsequently, the tube formation observed in these images was quantified using the Angiogenesis Analyser plugin within ImageJ software. These experimental procedures were repeated at least three times.

- 1. **Cell migration assay**

To assess the influence of tFNAs-miR22 on the migratory behavior of HUVECs, a wound healing assay was conducted. HUVECs were seeded in 6-well plates and incubated for 24 hours. Subsequently, the cells were divided into six groups and exposed to appropriate substances (control solution, tFNAs/miR22/tFNAs-miR22 at a concentration of 100 nmol·L-1, or AFL at a concentration of 1 mg·mL-1) in growth factor and FBS-free culture medium. Following this, a scratch was made using a sterile pipette tip, and images of the wound area were captured after incubation under normal oxygen conditions (37℃, 5%CO2) or low oxygen conditions (37℃, 1% O2, 5%CO2) for time intervals of 0, 24, and 48 hours using an Inverted Biologic Microscope (IX2-SL; Olympus Corporation). Statistical analysis was performed utilizing ImageJ software. Each experiment was replicated no less than three times.

1. **Oxygen-induced retinopathy (OIR) model**

The C57BL/6J mice used in this study were obtained from GemPharmatech Co., Ltd (Nanjing, China). The induction of oxygen-induced retinopathy (OIR) was performed in accordance with the methodology described in our previous publication. All animal experiments conducted in this study were approved by the Institutional Animal Care and Use Committee of Zhongshan Ophthalmic Center (Approval No: Z2023063).

1. **Intravitreal injection**

First, mice were anesthetized with 1% pentobarbital sodium (50mg·kg -1). Next, 1µL of AFL (10μg·μL-1), miR22 (10nmol·L-1) without transfection reagent, tFNAs (1μmol·L-1) or tFNAs-miR22 (1μmol·L-1) was injected into the vitreous cavity. The injections were carefully administered using a 33-gauge Hamilton syringe (Hamilton, USA) under a stereomicroscope (M620 F20, Leica Microsystems, France) to sidestep any potential damage to the lens. These injections took place at P12 in OIR mice. For the control, 1µL of a vehicle solution was injected intravitreally. Post-operation, a tobramycin eye ointment was applied as a preventative measure against potential infections.

1. **Measurement of retinal neovascularization (RNV)**

To assess the extent of retinal neovascularization (RNV), retinal images obtained from various treatments were randomly selected, labeled, and examined. The focus was on calculating the proportion of RNV area in relation to the total retinal area. Two independent reviewers, who were unaware of the treatment details, conducted all analyses. The presence of RNV was determined using a modified technique commonly employed in clinical trials^[12]^ and animal model studies^[13]^. ImageJ software was used to quantify both the RNV area, vaso-obliterated area and total retinal area. Absolute and percent ratios of the measured areas were used for statistical analysis.

1. **Statistical analysis**

The statistical analysis was conducted using SPSS Statistics 25.0, and the results from multiple experiments were presented as means ± standard deviation (SD). To assess significant differences between groups, a one-way ANOVA test was employed with P values below 0.05 indicating statistical significance. The number of repetitions and/or total animals involved can be found in either the figure legends or directly on the figures themselves
